# Cognitive Profiles are Better Predictors of Literacy Attainment Than Diagnostic Outcomes in Children with High ADHD Symptoms

**DOI:** 10.1007/s10803-024-06392-5

**Published:** 2024-06-16

**Authors:** Sinead Rhodes, Josephine N. Booth, Emily McDougal, Jessica Oldridge, Karim Rivera-Lares, Alexia Revueltas Roux, Tracy M. Stewart

**Affiliations:** 1 Clinical Brain Sciences, University of Edinburgh, Edinburgh, UK; 2 Moray House School of Education and Sport, University of Edinburgh, Edinburgh, UK; 3 Evidence Based Practice Unit, University College London and Anna Freud, London, UK; 4 School of Health and Wellbeing, University of Glasgow, Glasgow, UK; 5 School of Psychology and Neuroscience, University of Glasgow, Glasgow, UK

**Keywords:** ADHD, Cognition, Literacy, Heterogeneity, Diagnostic Threshold

## Abstract

We examined whether cognitive profiles or diagnostic outcomes are better predictors of literacy performance for children being considered for an ADHD diagnosis. Fifty-five drug naïve children (*M*_*age*_ = 103.13 months, *SD* = 18.65; 29.09% girls) were recruited from an ADHD clinical referral waiting list. Children underwent assessment of IQ, Executive Functions (EF) and literacy attainment. Hierarchical cluster analysis was used to generate subgroups of children using EF scores. Data were then grouped based on presence of a clinical ADHD diagnosis and the results compared. Grouping participants by profiles of cognitive test scores led to groups which also differed on literacy scores. However, categorising by whether children had received an ADHD diagnosis or not did not differentiate either cognitive tests scores or literacy scores. Cognitive performance, rather than children’s diagnostic outcomes, is more informative for identifying groups who differ in their literacy attainment which has important implications for remedial support.

Attention Deficit Hyperactivity Disorder (ADHD), characterised by persistent and pervasive inattention, hyperactivity, and impulsivity, is a common neurodevelopmental disorder. ADHD is estimated to affect 1–2% of children but an additional 5% fall below the threshold required for a clinical diagnosis and show difficulties arising from their symptoms (Karalunas & Nigg, 2020). Children with ADHD often show academic learning difficulties that begin to emerge as early as 3 years of age (Polanczyk et al., 2014) and they are at greater risk of adverse educational outcomes (Loe & Feldman, 2007). Academic learning difficulties in children can have far reaching and lifetime consequences, such as an increased likelihood of unemployment (Kuriyan et al., 2013) and mental health difficulties (Mammarella et al., 2016). It is crucial that these learning difficulties are well understood, and that appropriate educational interventions are applied. Academic learning difficulties in ADHD include both numeracy (Kanevski et al., 2022) and literacy (McDougal et al., 2022a, 2022b). There is evidence that literacy difficulties are associated with the ‘inattention’ dimension of ADHD symptoms (Plourde et al., 2015). In addition, ADHD diagnosis at 4 to 6 years of age has been shown to predict poorer literacy in adolescence after controlling for IQ (Massetti et al., 2008).

## ADHD and Literacy Attainment

Reading difficulties are evident from early in development in children with ADHD (Çelik et al., 2017; Miller et al., 2012). DuPaul et al. (2016) reported that over a third of children with ADHD had consistently poor reading achievement when their performance was measured across four different time points between the ages of 5 and 11 years. Accumulating evidence suggests that literacy difficulties are broad and extend beyond reading. For example, several studies have reported that children with ADHD show difficulties with spelling (Åsberg Johnels et al., 2014; Massetti et al., 2008; Silva et al., 2020). There is also evidence that children with ADHD have difficulties with written expression (Mayes & Calhoun, 2007a, 2007b). A recently published systematic review reported evidence across studies where compared to their peers, school-age pupils with ADHD have more significant difficulties in high order writing performance, which refers to writing quality and writing process, including planning and editing written work (Cheng, Coghill &Zendarski, 2022). This suggests the possibility that certain domains of cognitive functioning may be underlying these difficulties, and that further research is warranted to better understand and support them.

## The Role of Cognition

Researchers have identified a range of cognitive factors associated with literacy attainment (Cheng et al., 2022; Nouwens et al., 2020). These cognitive factors may underpin the development of difficulties with aspects of literacy in children with ADHD (McDougal et al., 2022a, 2022b). Executive functions (EFs) are higher-order cognitive processes used to organise our behaviour, such as working memory, planning, strategy use, organisational memory and attention flexibility, and have been highlighted due to their close affiliations with attentional control (Brocki et al., 2010) and as areas of particular difficulty in ADHD (Gau & Shang, 2010; Kofler et al., 2019; Miller et al., 2013; Rhodes et al., 2004, 2005; Rhodes et al., 2012). Conceptualisations of EF typically comprise inhibitory control, cognitive flexibility/set shifting, working memory (WM) and planning (Diamond, 2013; Miyake et al., 2000). The prominent working memory model by Baddeley and Hitch (1974) describes a capacity-limited central executive of the WM system that uses attentional processing for actively regulating, manipulating, and updating information in real time. The model specifies a phonological loop and visuospatial sketchpad that are responsible for storing modality-specific information in short-term memory in the absence of concurrent processing (Baddeley & Hitch, 1974; Cowan, 2011). Difficulties in these aspects of cognitive function in children with ADHD have been linked to poorer literacy outcomes (Åsberg Johnels et al., 2014; McDougal et al., 2022a, 2022b).

## Heterogeneity of Cognitive Function in Children with ADHD

Children with ADHD, however, differ from one another across a range of areas of functioning such as developmental trajectories, symptom presentation, co-occurrences and cognitive functioning (Luo et al., 2019). Marked within-group variability in cognitive function has been reported across several studies of children with ADHD (Coghill, 2010; Kofler et al., 2019). Coghill (2010) examined cognitive functioning in a large sample of drug naïve children with ADHD across nine cognitive tasks that tapped executive functions, alongside those that tapped more basic aspects of cognitive function such as short-term memory. Just under a fifth (18.7%) of the sample showed no significant difficulties on any of the tasks with 41% showing difficulties on executive function tasks and a further 40% only showing difficulties on cognitive tasks without a prominent EF component. Another study reported higher rates of difficulty on EF tasks but again profiles differed. Kofler et al. (2019) reported that 89% of the children with ADHD in their sample demonstrated difficulties in one or more aspect of EF, with 62% having a difficulty in working memory, 27% on an inhibitory control task, and 38% on an attention set shifting task. Just over half of the sample (54%) showed difficulties on one executive function task with around a third (35%) showing difficulties on two or three aspects of executive function measured. Collectively this research shows that children with ADHD differ in their specific cognitive profiles, and the level of difficulty they experience. The implications of this neurocognitive heterogeneity in children with ADHD for literacy remain unclear.

## Data-Driven Approaches

Data-driven analytical strategies have been used in the literature to identify distinct groups of children according to their cognitive profiles (Astle et al., 2019; Bathelt et al., 2018; Kofler et al., 2017; Nuñez et al., 2020; Roberts et al., 2017). For example, Astle et al. (2019) assessed cognition and academic learning in a large sample of 5- to 17-year-olds who had been referred for learning difficulties. One group of children had no age-related cognitive difficulties, and their academic learning attainment was in the typical range. In contrast they identified three other groups who had distinct cognitive profiles and diverse academic learning difficulties. One of these groups showed generalised cognitive difficulties and had extremely poor attainment in spelling, maths and reading, putting them in the bottom 5% of the population for performance. Another group had difficulties in phonological processing, in verbal short term memory and in working memory, broadly described as having phonological difficulties. The final group had significant difficulties in working memory including spatial working memory. Both the phonological difficulties group and the working memory difficulties group had significant difficulties in spelling, reading, and maths, but were similar to one another in these areas. Astle et al. (2019) reported that clinical diagnosis was not predictive of cognitive profiles, suggesting that a data driven cognitive profiling approach is more informative than grouping children according to traditional clinical diagnoses. Research that directly compares cognitive domains and academic abilities in children with and without confirmed diagnosis of ADHD is warranted to further address this question.

Existing studies have tended to examine a restricted range of aspects of literacy and/or cognitive function, making it difficult to ascertain cognition-literacy clusters according to the range of aspects known to be areas of difficulty for children with ADHD. In particular, the literature is lacking information about the broader aspects of literacy beyond word reading and spelling that are known to be problematic for children with ADHD (Mayes & Calhoun, 2007a, 2007b) and that are related to cognitive functions in these children (McDougal et al., 2022a, 2022b). Furthermore, some studies have been limited by not including children with ADHD who are drug naïve (e.g., Astle et al., 2019) as there is evidence that stimulant medication improves cognitive function (Coghill et al., 2007; Rhodes et al., 2004, 2006) even under 24-48h washout periods (Hawk et al., 2018; Kortekaas-Rijlaarsdam et al., 2019; Leo & Cohen, 2003; Powers et al., 2008). The current study set out to address these limitations and to add to the growing literature on data-driven approaches understanding academic learning, specifically literacy in ADHD. This will be beneficial for informing remedial support strategies which are generally only applied once a child receives a formal clinical diagnosis.

Our design, involving assessment of children on a National Health Service (NHS) ADHD waiting list and following these children until diagnosis, enables us to address an important question concerning early intervention. Children in the U.K. are on long NHS waiting lists for assessment of ADHD, typically 18 months upwards with some children waiting up to 3 years (Crane et al., 2018). Governments are aware of the difficulty this causes with initiating support and interventions. For example, a Scottish Government National Neurodevelopmental Specification (September, 2021) refers to the need for health and education professionals to “respond as early as possible to any indications that children, young people and their families/carers may need support” which “should not wait for diagnosis”. A James Lind Alliance Priority Setting Exercise indicated that identifying the ages and stages during which interventions are best introduced was a top prioritised question by young neurodivergent people, parents and health and education professionals (Lim et al., 2019). Previous research has mostly focused on cognitive and learning assessment of diagnosed children and given the current lengthy diagnostic waiting times it is difficult toascertain an answer to this question via studies with diagnosed children.

A lack of research makes it difficult to ascertain which professional is best placed to identify indicators of cognitive and learning needs in children not yet diagnosed and what would be a reliable indicator of those needs. There is some indication from the literature that professional referral alone is not sufficient to reliably identify those in need of cognitive and learning support. Astle et al. (2019) examined children referred by clinical or educational professionals for indications of ongoing problems in “attention”, “learning” or “memory” or “poor school progress”. The majority did not have a neurodivergent diagnosis (64%). Their cluster analysis revealed a group that showed age appropriate cognitive and learning performance and this group comprised a quarter of the sample (24.9%) indicating they did not need to be referred. In the current study, the cognitive and learning performance of children who have been accepted on to a clinical pathway following completion of the NHS ‘Choice’ appointment process will be examined. This process not only includes referral from a clinical or educational professional but information is also obtained around Neurodevelopmental symptoms from the parent and teacher. The parent also takes part in a brief interview (c. 1 h duration) with a member of the NHS Child and Adolescent Mental Health Services (CAMHS) team to clarify the information obtained. The current study design with assessment of children on a waiting list and subsequent follow up with diagnostic information will enable the examination of whether waiting list acceptance following the Choice appointment is indicative of the need for early support for all children. If our findings indicate no difference in diagnostic grouping this suggests there should be support at home and school at an earlier stage than is currently mandated.

The main aim of the current research study was to compare clinical diagnostic and cognitive profile grouping approaches to identify which are most informative for understanding children’s literacy skills. In the current study, children who were referred and on a waiting list for ADHD assessment took part. This recruitment approach enabled us to compare the cognitive function of children who received a diagnosis of ADHD with those who did not meet full criteria, identified from the same recruitment pool. This approach also meant that we were able to investigate cognitive profiles and literacy performance prior to any pharmacological intervention therefore exploring the “baseline” ADHD state, minimising the confounding effects of drug treatment. Taking a data-driven, bottom-up approach we grouped children using key theoretical EF areas that have been implicated as areas of difficulty in children with ADHD. Groups were compared on a comprehensive battery of tests assessing cognition and literacy skills. We also compared the same children using diagnostic category-based subgroups, depending on whether or not children received a clinical ADHD diagnosis after our evaluation.

The overall aim of the current study was to understand whether an EF performance data-driven approach was more informative regarding literacy skills of children with high symptoms of ADHD when compared to diagnostic driven groups. The specific research questions are: (1) Can the EF performance data of children with high ADHD symptoms be clustered into different cognitive profiles which could explain differences in literacy skills? (2) Are there differences in the literacy skills and cognitive performance of children with high ADHD symptoms depending on the presence or absence of a clinical diagnosis?

## Method

All the analyses in the present study were carried out after the study was pre-registered. The preregistration can be found at https://osf.io/enpbz/?view_only=0e95725db2774df2b646b75a19375716

## Participants

The sample consisted of 55 drug naïve children. They were recruited from the waiting list of children referred for ADHD assessment by NHS Child and Adolescent Mental Health Service (CAMHS) in the U.K. between 2019 and 2021. Children were aged 6 to 12 years (*M* = 103.13 months, *SD* = 18.65). Participants were chosen from the waiting list to ensure they were drug naïve (for ADHD related medications). The participants were recruited to a wider cohort study within the research group.

The following inclusion criteria were applied: (1) Children were on a waiting list for ADHD assessment by NHS CAMHS; (2) Participants were between 6 and 12 years old (to keep to UK primary school setting): (3) Participants were ADHD drug naïve; (4) All parents and children provided consent prior to participation; (5) Children with any additional co-occurring diagnoses were also included, apart from chromosomal conditions, as described below.

The exclusion criteria for data collection were as follows: (1) Primary language other than English; (2) Current/previous stimulant treatment; (3) A known chromosomal condition; (4) a known IQ score ≤ 70. The criteria for the data analysis led to the following further exclusions: (5) Scores within the typical range (≤ 60) on the Conners 3-Parent Diagnostic and Statistical Manual 5 (DSM-5) Inattention AND Hyperactivity-Impulsivity subscales (Conners, 2008) alongside no ADHD diagnosis by CAMHS; (6) An IQ score ≤ 70 (on both tests used); and (7) Missing data for more than half of the variables of interest.

Participants were given gift vouchers on agreeing to participate in recognition of time spent taking part in the research (£30). Favourable ethical opinion was granted from the Northwest Haydock Research Ethics Committee (Reference: 17/NW/0642). Descriptive and clinical information of the participants can be seen in Table 1.

**Table 1:** Descriptive and Clinical Information for Participants

	(N = 55)
Sociodemographic characteristics	
Age in months, Mean (SD)	103.13 (18.65)
Boys *n* (%)	39 (70.9%)
SIMD Quintiles *n* (%) *	Med = 3.50
1 (most deprived)	12 (21.8%)
2	10 (18.2%)
3	5 (9.1%)
4	8 (14.5%)
5 (least deprived)	19 (34.5%)
IQ	
WASI-II Full Scale IQ, Mean (SD)	96.43 (13.32)
BPVS, Mean (SD)	95.12 (12.33)
ADHD symptoms	
Conners ADHD Inattention T-Score, Mean (SD)	80.11 (11.17)
Conners ADHD Hyp/Imp T-Score, Mean (SD)	82.66 (10.12)
CAMHS diagnosis	
ADHD diagnosis *n* (%)	29 (52.7%)
ADHD + ASD diagnosis *n* (%)	5 (9.1%)
No ADHD diagnosis* n* (%)	21 (38.2%)
- ASD diagnosis *n* (%)	1 (1.8%)
- Awaiting ASD evaluation* n* (%)	6 (10.9%)
Co-occurring symptoms (above clinical cut-off) n (%) **	
Conners Oppositional Defiant Disorder (ODD)	40 (71.9%)
Conners Conduct Disorder* (CD)*	39 (70%)
Movement ABC Checklist *(DCD)*	13 (23.6%)
Autism Quotient ***	14 (25.5%)

*SIMD* Scottish Index of Multiple Deprivation; *CAMHS* Child and Adolescent Mental Health Service; *WASI-II* Wechsler Abbreviated Scale of Intelligence; *BVPS* British Picture Vocabulary Scale; *DCD* Developmental Coordination Disorder

*One child did not have SIMD information. **Number and percentage of participants who according to the manual, were at high risk of receiving a diagnosis. ***11 children scored above the threshold in the AQ10 (> 6) and 3 scored above the threshold in the AQ50 (> 76)

## Measures

### Clinical Profile Questionnaires

#### ADHD, ODD, CD Symptoms

The 110 item Conners 3-Parent assessed DSM-5 symptom criteria for the ADHD symptoms (Inattentive and Hyperactive/Impulsive), ODD (Oppositional Defiant Disorder), and CD (Conduct Disorder). A T-score ≥ 60 indicated clinically atypical symptom levels. This measure has been found by Kao & Thomas (2010) to have good internal consistency (Cronbach’s alpha = 0.90), test–retest reliability (*r* = 0.89) and interrater reliability (*r* = 0.84).

#### Movement Difficulties

The Movement ABC Checklist-2 (MABC-2; Schulz et al., 2011) is a parent-report measure of children’s movement difficulties in day-to-day settings. The MABC-2 is appropriate for children aged 5–12 years, with high classification agreement (80%-90%) to the Movement ABC Test (Schoemaker et al., 2012). The MABC-2 Checklist can be completed by parents as they observe the child in a wide variety of contexts. Higher scores indicated higher movement difficulties. This measure has been reported to have good internal consistency (α = 0.94; Schoemaker et al., 2012).

#### Autism Traits

Forty-one parents completed the Autism Spectrum Quotient-10 (AQ-10) to assess autism traits (Allison et al., 2012). A score of > 6 was used as a cut off point for high scores, requiring consideration of further assessment of Autism Spectrum Disorder (ASD) (sensitivity 0.95, specificity 0.97; Allison et al., 2012). Thirteen parents completed the Autism Spectrum Quotient-50 (AQ-50) to assess autism traits (Allison et al., 2012). A score of > 76 was used as a cut off point for high scores, requiring consideration of further assessment of ASD (sensitivity 0.95, specificity 0.97; Allison et al., 2012). The change of use of the AQ-10 to AQ-50 reflects that participants were taking part in a larger cohort study and a decision was made to use a more comprehensive measure.

#### Intellectual Functioning

The Wechsler Abbreviated Scale of Intelligence (WASI-II; Wechsler, 2011) was used to assess children’s global intellectual functioning. A Full-Scale IQ (*r* = 0.96) score was also calculated using all four subtests: Vocabulary, Similarities, Block Design, and Matrix Reasoning. The British Picture Vocabulary Scale (BPVS-III; Dunn et al., 2009) was used to provide an index of receptive vocabulary IQ. Children with a BPVS and a WASI-II Full-Scale IQ score ≤ 70 were deemed as potentially having an intellectual disability and were excluded from the study.

### EF Tasks

Participants completed four tasks from the Cambridge Neuropsychological Test Automated Battery (CANTAB®, Cognition, 2018) on a touch screen iPad and one working memory assessment (letters numbers sequencing task) from the Wechsler Intelligence Scale for Children (WISC-V; Wechsler, 2016).

#### Inhibitory Control

The Stop Signal Task examined children’s response inhibitory control. Participants responded to an arrow pointing in either left or right direction by pressing corresponding buttons. Responses had to be withheld if an auditory signal was heard. The key outcome measure was the stop signal reaction time (Stop Signal RT) in milliseconds (ms), which reflected the length of time between go stimulus and stop stimulus at which the children successfully withheld their response and did not select the button on 50% of trials.

#### Cognitive Flexibility

The Intra-Extra Dimensional task measured attentional set-shifting – the ability to flexibly switch attention between different stimuli characteristics. Participants selected abstract shapes and were prompted to learn rules regarding their choices via audio feedback. Once a rule was learned, the stimuli and/or rules changed, and participants had to shift attention to previously trivial stimulus attributes. The key outcome measure was the total number of times an incorrect stimulus was selected, adjusted for every stage (nine experimental stages in total) that was not reached (Intra-Extra Dimensional Errors/ID-ED errors).

#### Visuospatial WM Updating

The Spatial WM task examined visuospatial WM with updating. Participants were shown square 'boxes' and were asked to find a concealed token by looking in each box, with the caveat that once found, a token would not be hidden in the same box twice. The number of boxes increased from four, six, and eight items. The key outcome measure was the number of times participants incorrectly revisited a box in which a token was previously found (Spatial WM Between Search Errors).

#### Verbal WM Updating

The Letters Numbers Sequencing task (WISC-V) assessed verbal WM with updating. Participants listened to randomly presented letters and numbers and had to recite the numbers in ascending numerical order and the letters in alphabetical order. The total number of items increased from two to eight. The key outcome variable was children’s scaled score for the total number of trials (max = 30) for which the letters numbers sequence was correctly recited.

#### Planning

The Stockings of Cambridge task assessed children’s ability to monitor, evaluate, and update a sequence of planned moves. Participants copied a model pattern of three stacked coloured balls using a pre-specified minimum number of moves (either 2, 3, 4 or 5). The key outcome measure was the total number of problems solved in the minimum possible number of moves (Stockings of Cambridge Problems Solved).

### Literacy Skills

Literacy skills were measured using subtests of the Wechsler Individual Achievement Test—third U.K. edition (WIAT-III, Wechsler, 2017) and all results were reported in Standardised Scores (*M* = 100, *SD* = 15). The administration time of the following subtests varies with numerous factors, including age, academic strengths and weaknesses, test-taking style, and behaviour during testing (WIAT-III, Wechsler, 2017). The typical times for children between six and 12 years is reported. However, the number of items per test is not reported given the variability of rules across different subtests concerning the starting item based on the child's age and the specific discontinuation criterion of each test.

#### Basic Reading Composite

This composite score (*r* = 0.98) is comprised of Word Reading and Pseudoword Decoding subtests. The Word Reading subtest measured speed and accuracy of decontextualized word recognition. The child read aloud from a list of words that increased in difficulty. The list of words was read without a time limit. The examiner recorded the child’s progress after 30 s and continued administration until the discontinue rule was met or the last item was administered. The Pseudoword Decoding subtests measured the ability to decode nonsense words. The student read aloud from a list of pseudowords that increased in difficulty. The list of pseudowords was read without a time limit. The examiner recorded the child’s progress after 30 s and continued administration until the discontinue rule was met or the last item was administered. The administration of the Basic Reading Composite typically took between 5 and 10 min.

#### Reading Comprehension

This subtest (*r* = 0.81) measured untimed reading comprehension of various types of text, including informational text, how-to-passages, fictional stories, and advertisements. The child read passages aloud or silently. After each passage, the child orally responded to literal and inferential comprehension questions that were read aloud by the examiner. The administration of this subtest typically took between 8 and 30 min.

#### Spelling

This subtest (*r* = 0.94) measured written spelling of letter sounds and single words. The child heard each letter sound within the context of a word, and each word within the context of a sentence, and then they wrote the target letter sound or word. The administration of this tests typically took between four and 12 min.

#### Listening Comprehension

This subtest (*r* = 0.82) contained two components: Receptive Vocabulary and Oral Discourse Comprehension. Receptive Vocabulary measured listening. The child pointed to the picture that best illustrated the meaning of each word they heard. Oral Discourse Comprehension measured the ability to make inferences about, and remember details from, oral sentences and discourse. The child listened to sentences and passages and orally responded to comprehension questions. The administration of this subtest typically took between 10 and 22 min.

#### Oral Expression

This subtest (*r* = 0.82) contained three components: Expressive Vocabulary, which measured speaking vocabulary and word retrieval ability. The child said the word that best corresponded to a given picture and definition; Oral Word Fluency, which measured efficiency of word retrieval (i.e., how easily they could produce words) and flexibility of thought processes. The child named as many things as possible belonging to a given category (i.e., animals, colours) within 60 s; and Sentence Repetition, which measured oral syntactic knowledge and short-term memory. The child listened to sentences that increased in length and complexity and repeated each sentence verbatim. The administration of this subtest typically took between 8 and 17 min.

Testing was conducted across two to three sessions (each lasting around one hour) and typically took place either at home (first session) or at school (second and third sessions). At the first session, children completed the game-like CANTAB tasks in a counter-balanced order on an iPad, while the parent/carer completed the behaviour questionnaires. The second session was typically conducted at the child’s school in a quiet room. During the second and third sessions children completed assessments of literacy skills, IQ, and the verbal WM task. If a child’s individual needs required it, the assessments were spaced out over more sessions to ensure wellbeing and minimise effects of tiredness.

### Data Analysis

All analyses were conducted using IBM SPSS Statistics 24. Before analysis, data were checked for univariate outliers using criteria of a *z*-score > 3.29 (Field, 2018; Tabachnik & Fidell, 2013). One outlier was identified for the cognitive flexibility variable, produced by a coding error. This observation was removed. No other outliers were identified for the other cognitive or literacy variables. Multivariate outliers were also screened by using Mahalanobis distance scores for each respective analysis. Chi-square distributions of the Mahalanobis distance scores for the cognitive (*df* = 5) and literacy (*df* = 5) variables were all non-significant (*p* > 0.001).

Paediatric normative data for the CANTAB version used here was not available at the time of analysis, and so all raw scores were transformed into *z*-scores using participants’ age in years. The following variables were reverse scored so that higher scores indicated better performance: Stop Signal RT, ID-ED Errors, and Spatial WM Errors.

#### Cluster Analysis—Data Driven Cognitive Profiles

To address the first research question, we used a hierarchical clustering method applied to children’s EF *z*-scores to explore data-driven subgroups based on their cognitive profiles. Cluster analysis identifies homogenous groups of data objects based on similarities in characteristics within the group and dissimilarities between other groups (Tan et al., 2014). Cluster analysis has been used with small neurodivergent samples (e.g., Little et al., 2013; McDougal et al., 2020) and in ADHD research (e.g., Nuñez, et al., 2020; Roberts et al., 2017). Hierarchical cluster analysis is more appropriate for smaller data sets and facilitates more objective solutions than the alternative K-means clustering (Embrechts et al., 2013a, 2013b; Roberts et al., 2017). This uses agglomerative clustering, where each observation begins as its own cluster (Köhn & Hubert, 2015). The similarity distance between each cluster is calculated and similar observations are sequentially combined with each other until all observations are merged to produce a single large cluster. Ward’s method with Squared Euclidean distance (Ward, 1963) was employed which identifies pairs of clusters based on the criteria that the merger leads to a minimal possible increase in within-cluster variation (Dwyer et al., 2020, 2021). Ward’s method defines the distances between any two clusters as the magnitude of increase in the error sums of squares upon merging. Thus, Ward’s method merges clusters that minimise error sum of squares in each iteration (i.e., reducing the merging cost). Ward’s method consistently demonstrates good recovery of cluster structures and is less susceptible to noise than other methods (Everitt et al., 2011; Mojena, 1977).

##### Selection of Criterion Cluster Variables

In cases where the number of participants is small relative to the number of variables, the cluster classification may be weakened. The number of variables was therefore considered (Basagaña et al., 2013). Sample size recommendations suggest *N* = 2^m^ (i.e., 2 to the power of *m*) where *m* is the number of variables (Formann, 1984). Based on *n* = 55 (*N* = 32 minimum needed), five variables were deemed as appropriate to be used for clustering children based on their EF performance as well as for comparison of literacy skills (five variables) between cluster groups.. To check for multicollinearity, a collinearity diagnostic of absolute correlation values was used; all intercorrelations were below the required threshold (*r* < 0.8) and so were retained as individual cluster variables (Dormann et al., 2013; Tabachnik & Fidell, 2001). In line with previous studies, only children with complete data on cognitive criterion variables were included in the cluster analysis^1^ (Astle et al., 2019; Chen et al., 2019; McDougal et al., 2020; Vanbinst et al., 2015).

Theoretically informed EF (as detailed in the introduction section) criterion cluster variables included (1) Stop Signal RT (response inhibitory control); (2) Intra-Extra Dimensional Errors (set shifting); (3) Spatial WM Between Search Errors (visuospatial WM); (4) Letters Numbers Sequencing (verbal WM); and (5) Stockings of Cambridge Problems Solved (planning).

##### Identification of Clusters

In line with previous suggestions, the optimal cluster solution was informed using a visual inspection of the dendrogram figures and the more objective agglomeration coefficients (de Souza Salvador et al., 2019; Yim & Ramdeen, 2015). A sudden jump to a large coefficient between two consecutive stages indicated combination of potentially heterogeneous clusters and acted as a stopping point for the cluster process (Yim & Ramdeen, 2015). Another important consideration was that the emerging clusters are clinically relevant and include an adequate number of participants to allow for validation analysis (Bonafina et al., 2000). Once the clusters were identified, groups were characterised on their performance across each of the cognitive criterion variables.

##### Between Cluster Comparison

Multivariate Analysis of Variance (MANOVA)^2^ with follow-up univariate ANOVAs was used to compare the EF and literacy performance between the cluster groups. Pillai’s Trace was used as the criterion for determining effects, since the clusters did not have the same sample size. Post hoc Gabriel tests were used to contrast the groups in their performance, and in cases where homogeneity of variance assumption was violated, the Games-Howell post-hoc test was used (Field, 2018). MANOVAs were interpreted using partial omega squared (*ω*_*p*_^*2*^) which produces less bias in smaller samples than partial eta squared (Lakens, 2013; Okada, 2013). Partial omega squared effect size magnitudes were: 0.01 = small effect; 0.06 = medium effect; and large effect = 0.14 (Cohen, 1988).

##### Cluster Validation

To explore cluster validity, the identified cluster groups were compared on their age and SIMD using a Univariate ANOVA. A post hoc Gabriel test was used to contrast the groups in their performance (Field, 2018). The rates of ADHD diagnosis in each cluster were compared using Fisher’s exact test (Field, 2018). The rate of co-occurring disorder symptoms of ODD, CD, ASD and DCD was also examined to characterise the clusters.

## ADHD Diagnostic Subgroup Analysis

The second main analysis compared children with a clinical ADHD diagnosis to those without a clinical diagnosis who had high parent-rated ADHD symptoms (i.e., subclinical ADHD), on cognition, literacy, and age in months.

Independent sample t-tests were used to compare children with ADHD (*N* = 34) and subclinical ADHD (*N* = 21) on their cognitive and literacy performance. Normality within each group was checked using skewness and kurtosis z-scores using a cut-off of 1.96 (alpha level of *p* < 0.05) suitable for detecting non-normality in smaller samples (Kim, 2013; Tabachnik & Fidell, 2013). Non-parametric variant Mann–Whitney U test was used as an alternative to compare groups on variables that did not meet normality assumptions (Field, 2018). Effect size magnitudes for this analysis were calculated using Hedges *g* (0.2 = small effect, 0.5 = medium effect, 0.8 = large effect). For the non-parametric Mann Whitney U tests effect sizes were calculated using r (0.1 = small effect, 0.3 = moderate effect, and 0.5 = large effect; Field, 2018).

## Results

### Cluster Analysis—EF Data-Driven Clusters

#### Cluster Identification

Initial inspection of the dendrogram indicated a three or potentially five cluster solution (*Supplementary Material 1*). The agglomeration coefficient schedule also indicated a sudden jump at the 4–5 cluster combination and then again at 2–3 cluster combination (*Supplementary Material 2*). As such, 2, 3, 4, and 5 cluster solutions were considered, with the 3-cluster solution generating the most homogenous and interpretable subgroupings.

#### EF Characteristics of Clusters

**Fig. 1:**
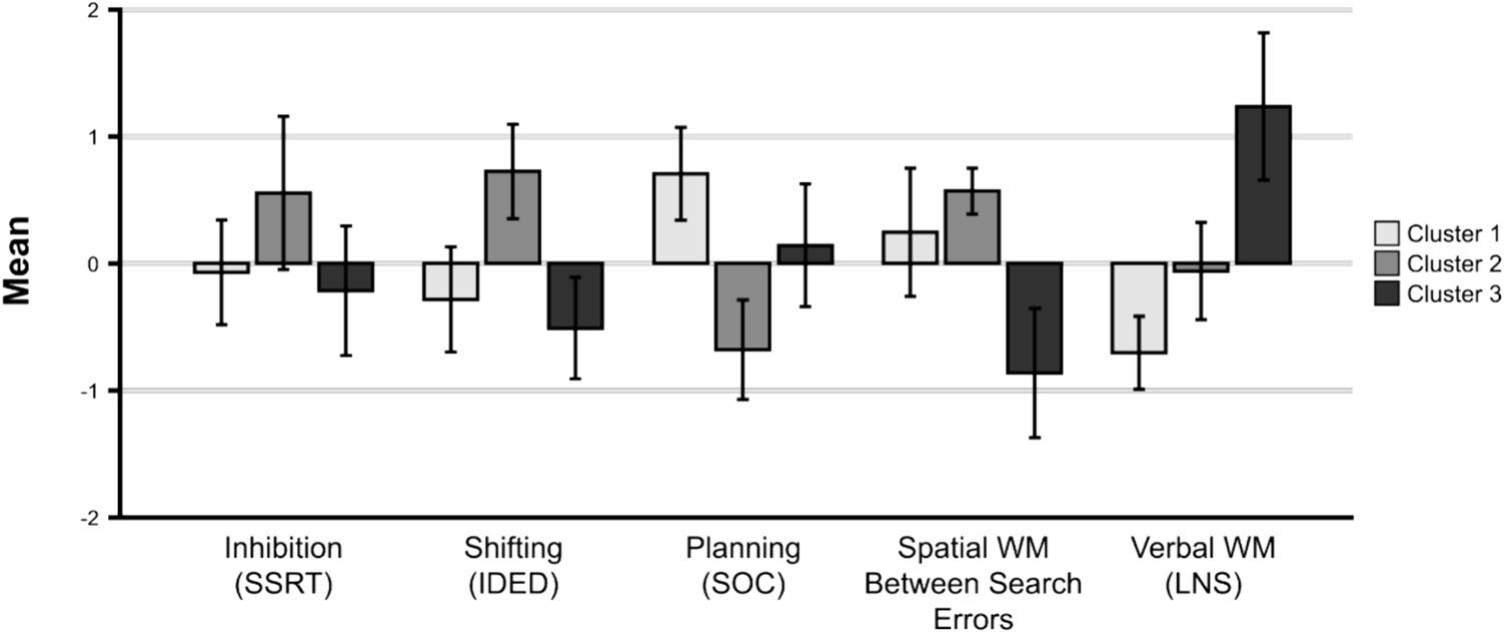
Performance of Clusters on Criterion EF Variables. SSRT Stop Signal Reaction Time; IDED Intra-Extra Dimensional; SOC Stockings of Cambridge; Spatial Working Memory; LNS Letters Numbers Sequencing

Performance of each cluster on the EF criterion variables is illustrated in Fig. 1. The descriptive statistics for each resulting cluster are provided in Table 2. The MANOVA revealed that the groups significantly differed in performance on the EF criterion variables, Pillai’s Trace = 1.440, *F*(10,72) = 18.49, *p* < 0.001, η_p_^2^ = 0.72. Separate univariate ANOVAs revealed significant differences between the group clusters on shifting (*p* = 0.000, *ω*^*2*^_*p*_ = 0.34), spatial WM (*p* = 0.000, *ω*^*2*^_*p*_ = 0.35), verbal WM (*p* = 0.000, *ω*^*2*^_*p*_ = 0.55), and planning (*p* = 0.000,* ω*^*2*^_*p*_ = 0.42). The difference in inhibitory control scores was not statistically significant (*p* = 0.070) and the effect size was small *(ω*^*2*^_*p*_ = 0.08). Significance values and effect sizes of the univariate group comparisons can be found in Supplementary Material 3.

**Table 2:** Descriptive Data and Results of ANOVA Comparisons Between EF Clusters (Cognition and Literacy Performance)

	Cluster 1 N = 17		Cluster 2 N = 15		Cluster 3 N = 10			*ANOVA*			Group Contrasts
	Mean	SD	Mean	SD	Mean	SD	*F* (2, 39)		*p*	*ω*^*2*^_*p*_	
Cognition (Criterion EF variables)											
Stop Signal Reaction Time	− .093	.828	.549	1.09	− .220	.7110	2.843		.070	.081	NS
Intra-Extra Dimensional Errors	− .283	.820	.733	.668	− .512	.553	11.863		< .000	.341	2 > 1, 1 = 3, 2 > 3
Spatial WM Between Search Errors	.248	.984	.581	.314	− .875	.691	12.306		< .000	.350	1 = 3, 1 > 2, 3 > 2
Letter Number Sequencing Standardised Score	− .709	.548	− 051	.696	1.25	.808	27.075		< .000	.554	3 > 1,3 > 2,2 > 1
Stockings of Cambridge Problems Solved	.716	.705	− .685	.699	.149	.680	16.154		< .000	.419	1 > 2,1 = 3,3 > 2
Literacy											
Basic Reading Composite Score	86.59	18.91	91.43	25.02	104	11.30	2.426		.102	.064	NS
Listening Comprehension	97.59	14.53	99.64	13.90	109.30	16.41	2.084		.138	.049	NS
Reading Comprehension	86.71	12.70	87.21	10.93	101.30		6.189		.005	.198	3 > 1, 3 > 2, 1 = 2
Spelling	82.65	14.97	85.78	12.44	97.3	7.76	4.334		.020	.137	3 > 1
Oral Expression Composite Score	90.59	12.61	98.79	14.49	106	13.66	4.239		.022	.134	3 > 1
Cluster differences											
Age	101.29	17.96	106.33	18.09	108.40	22.73	.507		.607	− .024	
SIMD	2.88	1.83	2.60	1.45	3.80	1.62	1.647		.206	.030	

*Stop Signal Reaction Time Inhibition; Intra-Extra Dimensional Set Shifting; Letters Numbers Sequencing Verbal WM updating; Stockings of Cambridge Planning; Spatial Span Reverse Visuospatial WM (w/o updating); Spatial Span Forwards Visuospatial STM; Delayed Matching to Sample Delayed Visuospatial Recognition Memory*

#### Performance and Characterisation of Clusters on Criterion EF Variables

##### Cluster 1: Low Verbal WM

Cluster 1 was characterised by low verbal WM and high planning. Cluster 1 had significantly lower verbal WM scores on the Letter Number Sequencing task than Cluster 2 (*p* < 0.025, *g* = 1.056) and Cluster 3 (*p* < 0.000, *g* = 2.998). Cluster 1 had significantly higher planning scores than Cluster 2 (*p* < 0.000, *g* = 1.997), and higher spatial WM than Cluster 3 (*p* < 0.001, *g* = 1.262). No substantive differences were found for the other variables.

##### Cluster 2: Low Planning & High Shifting

Cluster 2 scored lower on EF planning task but highest in attentional shifting. They solved fewer planning problems on the Stockings of Cambridge task than Cluster 1 (*p* < 0.000, *g* = − 1.997) and Cluster 3 (*p* = 0.017, g = − 1.205). However, they made a lower number of shifting errors on the Intra-Extra Dimensional task than Cluster 1 (*p* < 0.000, *g* = 1.349) and Cluster 3 (*p* < 0.000, *g* = 1.99). Cluster 2 also made less search errors on the Spatial WM task than Cluster 3 (*p* < 0.000, *g* = 2.930).

##### Cluster 3: Low Spatial WM & High Verbal WM

Children in Cluster 3 were distinguished by an overall lower EF performance than the other groups except for verbal WM. Children in Cluster 3 had higher scores on the Verbal WM task than Cluster 1 (*p* < 0.000, *g* = 2.999) and Cluster 2 (*p* < 0.000,* g* = 1.756). Furthermore, Cluster 3 had more errors in the spatial WM task than Cluster 1* (p* < 0.001, *g* =− 1.262) and Cluster 2 (*p* < 0.000, *g* =− 2.930).

##### Cluster Difference by Age and SIMD

The characteristics of participants in each of the EF clusters were examined by comparing groups on age and socioeconomic status with a Univariate ANOVA. The univariate ANOVA revealed no significant differences between the groups in age or socioeconomic status. The descriptive statistics for each cluster’s Age, SMID and statistical univariate ANOVA results are provided in Table 2.

##### Rates of ADHD and Co-occurring Symptoms between Clusters

Rates of clinical ADHD diagnosis in Cluster 1 (*N* = 10), Cluster 2, (*N* = 10) and Cluster 3 (*N* = 5) were similar. Fisher’s exact test showed that there was no significant association between cluster membership and receipt of a clinical ADHD diagnosis (*p* = 0.674) Table 3 shows descriptive data of ADHD diagnosis rates and co-occurring symptoms per cluster for further characterisation.

**Table 3:** Cluster Descriptive Characteristics and ADHD Symptoms and Co-occurrences per Cluster

Cluster characterisation	Cluster 1 N = 17			Cluster 2 N = 15			Cluster 3 N = 10			
	Mean		SD	Mean		SD	Mean		SD	
Age (months)	101.29		17.96	106.33		18.09	108.40		22.73	
SIMD	2.88		1.83	2.60		1.45	3.80		1.62	
CAMHS ADHD Diagnosis	N		%	N		%	N		%	
*Clinical ADHD*	10		58.8%	10		66.7%	5		50%	
*Subclinical ADHD	7		41.2%	5		33.3%	5		50%	
ADHD symptoms and co-occurrences	Mean	SD	Above threshold N (%)	Mean	SD	Above threshold N (%)	Mean	SD		Above threshold N (%)
Conners ADHD Inattention	76.81	13.32	14 (94.1%)	79.67	11.93	15 (100%)	82.80	8.92		10 (100%)
Conners ADHD Hyperactivity-Impulsivity	81.50	9.74	16 (94.1%)	82.20	10.13	15 (100%)	82.60	13.95		9 (90%)
Conners Oppositional Defiant Disorder	79	14.36	13 (76.4%)	72.40	18.41	9 (59.9%)	78	17.17		8 (80%)
Conners Conduct Disorder	72.94	14.85	13 (76.4%)	67.07	16.95	9 (59.9%)	68.50	16.83		7 (70%)
**Autism Quotient	–	–	3 (18%)	–	–	6 (40%)	–	–		1 (10%)
Movement ABC	13.47	13.93	7 (41.2%)	20.80	14.26	10 (66.7%)	15.70	16.96		6 (60%)

*Sub-clinical (rather than no ADHD) is used to refer to these children as they have high ADHD symptoms on parent rating scales and passed the choice assessment to be included for full clinical assessment. **Means and standard deviations are not shown due to two different versions of the AQ scale used

#### Literacy Performance Between Clusters

**Fig. 2:**
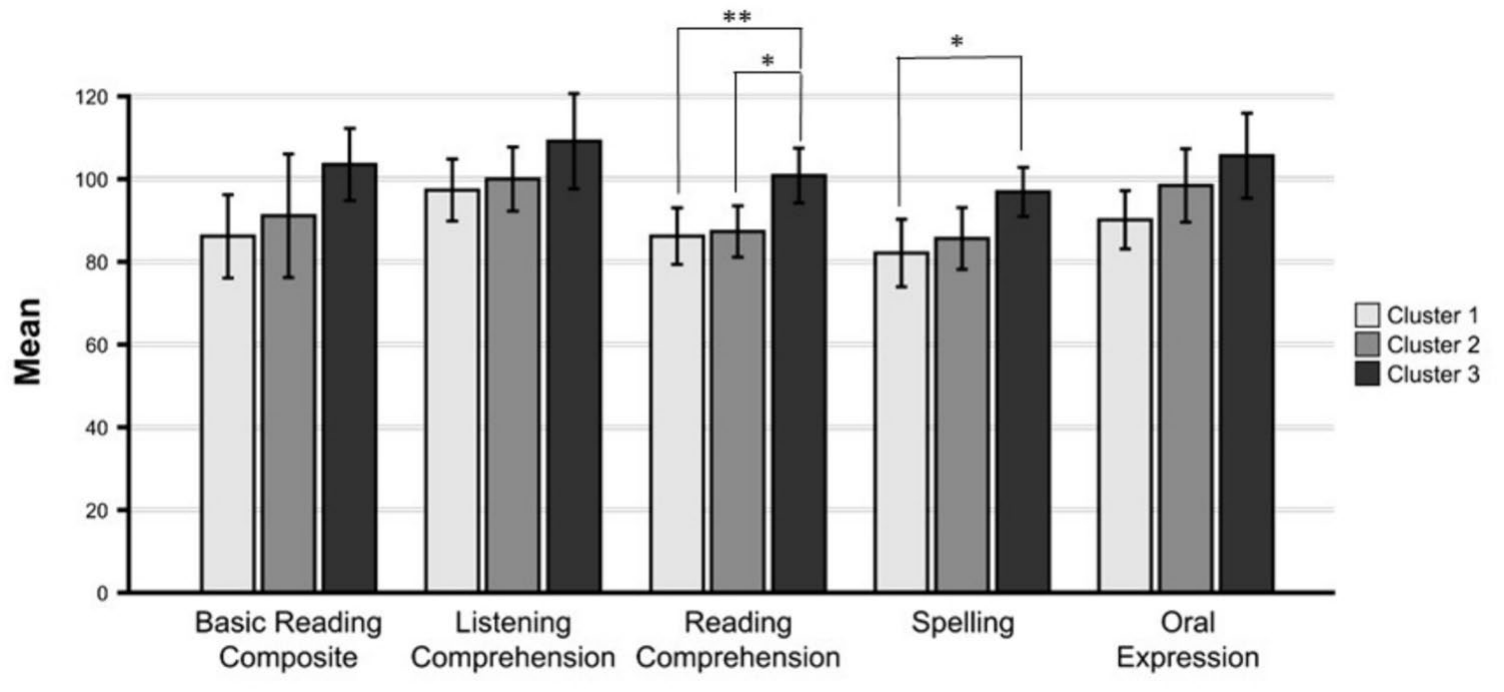
Performance of EF Clusters on Literacy Skills. Error bars are 95% Confidence Intervals

A MANOVA revealed no significant differences between the clusters in the literacy scores: Pillai’s Trace = 0.367 *F*(10,70) = 1.575, *p* = 0.132, η_p_^2^ = 0.184. However, differences were found in pairwise comparisons for Reading Comprehension, Spelling, and Oral Expression. Separate univariate ANOVAs revealed significant differences between the group clusters on Reading Comprehension (*p* = 0.005, *ω*^*2*^_*p*_ = 0.198), Spelling (*p* = 0.020, *ω*^*2*^_*p*_ = 0.137), and Oral Expression (*p* = 0.022,* ω*^*2*^_*p*_ = 0.134). The mean values are in Table 2. There were no significant differences in Basic Reading Composite scores (*p* = 0.102) or in Listening Comprehension (*p* = 0.138). Significance values and effect sizes of the univariate group comparisons can be found in Supplementary Material 3. Figure 2 shows the mean scores in the literacy skills by cluster.

##### Reading Comprehension

Children in Cluster 3 (Low spatial WM and high verbal WM) obtained higher scores at Reading Comprehension than children in Cluster 1 (Low Verbal WM; *p* = 0.007, *g* = 1.28) and Cluster 2 (Low planning and high shifting; *p* = 0.013, *g* = 0.042). There were no differences in Reading Comprehension between children in Cluster 1 and children in Cluster 2 (*p* = 0.999).

##### Spelling

Children in Cluster 3 (Low spatial WM and highest verbal WM) obtained higher scores than children in Cluster 1 (Low Verbal WM; *p* = 0.019, *g* = 1.14). There were no significant differences between children in Cluster 3 and children in Cluster 2 (*p* = 0.105) or between children in Cluster 1 and children in Cluster 2 (*p* = 0.999).

##### Oral Expression

Children in Cluster 3 (Low Verbal WM) obtained higher scores than children in Cluster 1 (Low Verbal WM; *p* = 0.021, *g* = 1.186). There was no significant difference between children in Cluster 3 and children in Cluster 2 (Low planning and high shifting; *p* = 0.617), or children in Cluster 1 and Cluster 2 (*p* = 0.304).

## ADHD Diagnostic Subgroup Profiles

The results of the diagnostic subgroup comparisons are presented in Tables 4 and 5.

**Table 4:** Descriptive Data and Results of Diagnostic Contrasts (Cognition, Age, SIMD, Literacy Performance, and Symptoms)

	ADHD			Subclinical ADHD		Group contrasts				
	N	Mean	SD	N	Mean	SD	t/U^a^	*p*		*g/r*
Cognition										
Stop Signal Reaction Time	32	− .149	.964	21	.226	.878	− 1.43		.159	.402
Intra-Extra Dimensional Errors	34	.008	1.056	21	− .012	.748	.077		.939	.021
Spatial WM Between Search Errors	33	.061	.965	21	− .096	.918	.603		.549	1.08
Letters Numbers Sequencing	28	− .055	.961	17	.090	1.084	− .467		.643	.143
Stockings of Cambridge Problems Solved	34	− .169	.914	21	.273	.946	− 1.72		.091	.477
Age & SIMD										
Age (months)	34	101.35	16.040	20	106	22.372	− 1.199	.236		.331
SIMD	34	3.35	1.756	20	3	1.376	.769	.445		.242
Literacy										
Basic Reading Composite Score	33	95.28	18.61	21	93.14	25.25	.448	.656		.125
Listening Comprehension	34	102.38	2.67	21	100.57	15.53	.419	.677		.116
Reading Comprehension	33	91.51	2.03	20	92.10	13.91	− .164	.870		.047
Spelling	33	88.09	14.33	21	84.28	14.17	.955	.344		.266
Oral Expression Composite Score	33	100.03	14.11	20	95.15	15.36	1.180	.243		.334

*Stop Signal Reaction Time* Inhibition*; Intra-Extra Dimensional* Set Shifting*; Letters Numbers Sequencing* Verbal WM Updating; *Stockings of Cambridge* Planning; *Spatial Span Reverse* Visuospatial WM (w/o updating)*; Spatial Span Forwards* Visuospatial STM; *Delayed Matching to Sample* Delayed Visuospatial Recognition Memory

**Table 5:** ADHD Symptoms and Co-occurrences per Diagnostic subgroup

ADHD symptoms and co-occurrences	ADHD			Subclinical ADHD		
	Mean	SD	Yes N(%)	Mean	SD	Yes N(%)
Conners ADHD Inattention	81.59	15.521	31(91.2%)	84.10	8.706	21(100%)
Conners ADHD Hyperactivity-Impulsivity	84.69	7.672	32(94.1%)	84.14	10.451	20(95.2%)
Conners Oppositional Defiant Disorder	74.63	16.018	23(67.8%)	79.71	16.022	17(80.9%)
Conners Conduct Disorder	68.78	16.303	21(62.1%)	73.24	12.957	18(85.7%)
Autism Quotient	-	-	6(17.6%)	-	-	8(38.1%)
Movement ABC	16.30	12.948	18(52.9%)	21.81	16.627	14(66.7%)

*The column “Yes” shows the number and percentage of children who scored above the threshold, and thus are considered at risk of being diagnosed*

### Cognition

There were no statistically significant differences (all *p*-values > 0.05) between children with and without a clinical ADHD diagnosis on any of the cognitive outcome variables (*g* ranged from 0.021 to 1.08).

### Literacy

There were no statistically significant differences (all *p*-values > 0.05) between children with and without a clinical ADHD diagnosis on any of the literacy variables (*g* ranged from 0.047 to 0.334).

### Diagnostic Subgroup Difference by Age & SIMD

There were no statistically significant differences (all *p*-values > 0.05) between children with and without a clinical ADHD diagnosis on age (*g* = 0.331) and SIMD (*g* = *0.242*).

## Discussion

The current study set out to examine whether a data-driven or a diagnostic approach was more effective in understanding the literacy performance of children with high parent-rated ADHD symptoms. A three-cluster solution was chosen to characterise the full sample on the basis of identifying the most homogenous and interpretable subgroupings; these clusters were significantly different in their EF profiles. The clusters differed in relation to attention set-shifting, spatial working memory, verbal working memory, and planning. Cluster 1 was characterised by low verbal working memory; cluster 2 was characterised by low planning and high shifting; and cluster 3 by low spatial working memory and this group had the highest verbal working memory performance. The clusters differentially predicted literacy skills with significant differences observed in relation to reading comprehension, spelling, and oral expression.

Working memory function, in particular, was important for literacy relationships. Better verbal working memory function was shown to be characteristic of superior performance in reading, spelling and oral expression. Working memory has previously been highlighted as important for reading in studies with typically developing children (Nouwens et al., 2020) and for children with ADHD (McDougal et al., 2022a, 2022b). Our findings support this and suggest the relationship extends to other aspects of literacy namely spelling and oral expression. Rates of ADHD diagnosis were shown to be similar amongst the three clusters. The study findings show that a data driven approach is effective for understanding literacy attainment in ADHD.

There were no significant differences in EF performance between the children with high symptoms of ADHD who did receive a diagnsis and those who did not. There was also no significant difference in literacy performance between children with high symptoms of ADHD who did and did not receive a diagnosis. These findings suggest that a data driven rather than a diagnostic approach is more effective for understanding literacy difficulties in children with ADHD, supporting previous work in this area (Astle et al., 2019; Bathelt et al., 2018). The findings also suggest that relations between cognition and literacy extend beyond reading and spelling, to also include oral expression. Differential relations between aspects of cognition and literacy suggest that support tailored to children’s individual cognitive profile rather than tailored to their diagnostic label is an important grouping shift and can be beneficial for ensuring children with ADHD have optimal academic outcomes.

Importantly, these findings show that children with high, but sub-clinical threshold, ADHD symptoms can also have difficulties with cognitive function and academic learning performance. Our study design enabled us to examine the cognitive and literacy profile of children on the same clinical pathway at the same time pre-diagnosis, comparing those who later met the diagnostic threshold for ADHD after clinical assessment, with those who did not. Children who have high ADHD symptoms but do not meet the diagnostic threshold appear to show the same extent of thinking and learning difficulties as their diagnosed peers. These findings have implications for understanding and supporting children in schools who may show high symptoms of a neurodevelopmental disorder but do not meet diagnostic criteria. Previous findings have indicated that professional referral alone is not sufficient to indicate cognitive and learning support need with a quarter of children in the sample showing age appropriate performance (Astle et al., 2019). Our findings suggest that having high ADHD symptoms and passing the threshold for being accepted onto a waiting list for assessment (NHS Choice procedure) in itself is indicative of difficulties in thinking and learning that need support. The findings of our research show that support need not wait on diagnosis before it is provided. This assessment involving a professional referral, symptom information obtained from parents and teachers accompanied by a brief parent interview is sufficient to indicate need for cognitive and learning support. Our findings suggest that support for cognitive and learning difficulties should be provided as soon as a child is accepted onto a waiting list, if already not in place.

## Limitations

One potential limitation is the relatively small sample size of the present study. However, power analysis revealed that a sample of n = 32 was required to conduct cluster analysis with 5 variables; our sample of n = 55 generated sufficient power and we can have confidence in the findings presented. A further limitation relates to the range of measurement tasks included. While a comprehensive assessment package brings a benefit of enhancing our understanding, undertaking large numbers of assessments can place a burden on participants and their attentional focus. Tasks were completed in two to three sessions and researchers responded to the individual needs of participants and provided extra breaks as required. As the order of the cognitive tasks was spread across sessions and completed in variable order, we can also have confidence that fatigue did not adversely impact results. An inclusive approach including children with common co-occurrences e.g., autism was taken in the current study which we believe is a key strength of the study. We did not screen though for mental health diagnoses (e.g. = depression or anxiety), which themselves are associated with cognitive difficulties (Mammarella et al., 2016). We were mindful of overburdening participants with extensive assessments and questionnaires.

## Implications and Conclusions

The findings from the current study suggest that each child should be provided with tailored support relevant to their needs from the point their difficulties have been identified and they join a waiting list, rather than being dependent on a clinical diagnosis. In practice, the findings indicate that literacy difficulties in children with high ADHD symptoms may be supported by strategies based upon a child’s individual cognitive profile and needs. For example, a child within the cluster one sample may be supported in their literacy skills by implementing verbal memory supports, whereas a child in cluster two could be best supported with planning strategies. The findings highlight the need for further research on tailored cognitive interventions and use of individualised support strategies to facilitate literacy attainment abilities in children with high ADHD symptoms.

## Footnotes

1 This meant excluding 13 participants from the analysis leaving a sample size of 42. This was still beyond the variable criterion threshold.

2 A MANOVA was selected as it reduces Type 1 error rates and accounts for the relationship among the dependent variables (Field, 2018).
